# Open multi-center intracranial electroencephalography dataset with task probing conscious visual perception

**DOI:** 10.1038/s41597-025-04833-z

**Published:** 2025-05-23

**Authors:** Alia Seedat, Alex Lepauvre, Jay Jeschke, Urszula Gorska-Klimowska, Marcelo Armendariz, Katarina Bendtz, Simon Henin, Rony Hirschhorn, Tanya Brown, Erika Jensen, Csaba Kozma, David Mazumder, Stephanie Montenegro, Leyao Yu, Niccolò Bonacchi, Diptyajit Das, Kyle Kahraman, Praveen Sripad, Fatemeh Taheriyan, Orrin Devinsky, Patricia Dugan, Werner Doyle, Adeen Flinker, Daniel Friedman, Wendell Lake, Michael Pitts, Liad Mudrik, Melanie Boly, Sasha Devore, Gabriel Kreiman, Lucia Melloni

**Affiliations:** 1 Department of Neurology, New York University Grossman School of Medicine, New York, NY 10016 USA; 2 Neural Circuits, Consciousness and Cognition Research Group, Max Planck Institute for Empirical Aesthetics, Frankfurt am Main, 60322 Germany; 3 Donders Institute for Brain, Cognition and Behavior, Radboud University Nijmegen, Nijmegen, 6500 HB the Netherlands; 4 Department of Psychiatry, University of Wisconsin-Madison, Madison, WI 53719 USA; 5 Boston Children’s Hospital, Harvard Medical School, Boston, MA 02115 USA; 6 Center for Brains, Minds and Machines, Cambridge, MA 02139 USA; 7 Harvard Medical School, Boston, MA 02115 USA; 8 Sagol School of Neuroscience, Tel Aviv University, Tel Aviv, 6997801 Israel; 9 Newcastle University, Newcastle upon Tyne, NE4 5TG UK; 10 Department of Biomedical Engineering, New York University School of Engineering, New York, NY 11201 USA; 11 Champalimaud Research, Lisbon, 1400-038 Portugal; 12 William James Center for Research, ISPA - Instituto Universitario, Lisbon, 1149-041 Portugal; 13 Comprehensive Epilepsy Center, NYU Langone Health, New York, NY 10016 USA; 14 Department of Neurosurgery, NYU Langone Health, New York, NY 10016 USA; 15 Department of Neurology, University of Wisconsin-Madison, Madison, WI 53726 USA; 16 Psychology Department, Reed College, Portland, OR 97202 USA; 17 School of Psychological Sciences, Tel Aviv University, Tel Aviv, 69978 Israel; 18 Program for Brain, Mind, and Consciousness, Canadian Institute for Advanced Research, Toronto, Ontario Canada; 19 Predictive Brain Department, Research Center One Health Ruhr, University Alliance Ruhr, Faculty of Psychology, Ruhr University Bochum, Bochum, 44801 Germany

**Keywords:** Consciousness, Object vision

## Abstract

We introduce an intracranial EEG (iEEG) dataset collected as part of an adversarial collaboration between proponents of two theories of consciousness: Global Neuronal Workspace Theory and Integrated Information Theory. The data were recorded from 38 patients undergoing intracranial monitoring of epileptic seizures across three research centers using the same experimental protocol. Participants were presented with suprathreshold visual stimuli belonging to four different categories (faces, objects, letters, false fonts) in three orientations (front, left, right view), and for three durations (0.5, 1.0, 1.5 s). Participants engaged in a non-speeded Go/No-Go target detection task to identify infrequent targets with some stimuli becoming task-relevant and others task-irrelevant. Participants also engaged in a motor localizer task. The data were checked for its quality and converted to Brain Imaging Data Structure (BIDS). The de-identified dataset contains demographics, clinical information, electrode reconstruction, behavioral performance, and eye-tracking data. We also provide code to preprocess and analyze the data. This dataset holds promise for reuse in consciousness science and vision neuroscience to answer questions related to stimulus processing, target detection, and task-relevance, among many others.

## Background & Summary

Since the 1930s, intracranial electroencephalography (iEEG) has been the gold standard in identifying epileptogenic areas for surgical resections and other targeted treatment techniques^1^. IEEG recording strategies vary across centers, ranging from the use of cortical grid and strip electrodes (Electrocorticography, ECoG) to the exclusive use of stereotactic depth electrodes (Stereoelectroencephalography, sEEG), or a combination of both^2^. Using iEEG, clinicians and scientists alike can characterize and localize signals acquired directly from the brain. During lulls in clinical care, willing research participants may choose to volunteer for studies that analyze signals acquired via iEEG^3^. Beyond their significant clinical utility, these signals provide a valuable tool for investigating the neurophysiology of cognition^4–7^.

These recordings, completed as part of the clinical standard of care, are rare, primarily due to the small number of patients requiring this procedure to begin with^7,8^. In addition, the clinical nature of iEEG data necessitates taking greater precautions when sharing them openly to prioritize the privacy of the participants, compared to more typical non-invasive techniques such as functional magnetic resonance imaging (fMRI) or scalp electro- and magneto-encephalography (EEG and MEG) used to investigate cognitive processes. However, iEEG recordings offer much better spatio-temporal resolution over non-invasive neuro-imaging and non-invasive electrophysiology^9^, which makes them critical to accelerating cognitive neuroscience research^4^. These circumstances emphasize the importance of creating larger datasets for public availability and reuse, which is further amplified by the difficulty in collecting these data.

Importantly, data must be shared with sufficient background information such that they can be easily reused by other researchers, in line with the FAIR (Findable, Accessible, Interoperable, Re-usable) principles^10^. Achieving this goal is particularly challenging in the case of iEEG recordings due to the high diversity and variability of data types associated with iEEG^3^ as well as experimental setups. The placement of the iEEG electrodes is solely determined based on clinical needs and therefore varies greatly from participant to participant. Furthermore, a single participant dataset consists of electrophysiological recordings, neuro-imaging data, electrode localization, behavioral files, clinical information and additional metadata files. Therefore, there can be significant variability in file format, labeling and directory structure from center to center for each of the data types associated with iEEG recordings^11^. In addition, the data are usually collected within the patient hospital bedroom, leading to a high variability in the experimental conditions under which the data were collected, which is rarely documented. Finally, there is ample variability between participants, associated with their age, disease diagnosis and clinical history. With this wealth and diversity of relevant information comes the challenge of effectively organizing data to enable reusability. Brain imaging data structure (BIDS)^12^ provides a solution to some of these issues by providing a standardized nomenclature for file naming, organization, and documentation. BIDS defines file formats for each type of data, naming conventions for each file, folder structure for all the data, as well as which data and metadata are mandatory or optional for an iEEG data set^11^. However, the BIDS specifications are currently under-specified for documenting the clinical information of the participants^3^, but also the experimental context in which the research took place.

In this article, we directly address these challenges by openly sharing a comprehensive iEEG dataset from 38 participants^13^. The experimental paradigm and associated data shared here were collected as part of a large-scale international adversarial collaboration testing key contradictory predictions of two prominent theories of Consciousness:^14^ Global Neuronal Workspace Theory (GNWT)^15^–^19^ and Integrated Information Theory (IIT)^20^–^23^. In this study, participants were exposed to foveally presented, suprathreshold stimuli varying in their category (faces, objects, letters and false fonts) orientation (left view, right view, front view), and duration (0.5, 1.0, and 1.5 seconds). Those manipulations aimed to model the richness of conscious perception, and in doing so enabled testing key theoretical predictions of GNWT and IIT with respect to the brain areas claimed necessary for consciousness. Critically, to dissociate neural profiles of responses associated with consciousness from other confounding factors related to, for instance report and/or task performance, participants were asked to detect infrequent targets from two stimulus categories while the other categories remained task-irrelevant (counterbalanced across blocks). As such, the same categories were sometimes task-relevant, while in other blocks task-irrelevant. In contrast, both stimulus orientation and duration were always task-irrelevant. Multiple brain imaging modalities were collected in different groups of human participants. In addition to the iEEG dataset shared here, functional magnetic resonance imaging (fMRI) and simultaneous electroencephalography and magnetoencephalography (MEG) were also collected in an independent sample of neurotypical participants. These additional datasets will be shared in separate publications.

Beyond its primary goal of adjudicating between these theories, the paradigm enables the investigation of a range of other cognitive and perceptual phenomena. For example, by including distinct categories of visual stimuli, it allows for the exploration of how different types of visual information are processed and how they are affected by task manipulations. Stimuli are presented for varying durations, providing an opportunity to examine how the temporal characteristics of a stimulus influence the persistence and continuity of conscious experience, thereby shedding light on the dynamics of sustained perception^24–27^. The inclusion of orientation in the experimental paradigm allows for the examination of how neural representations of visual stimuli remain consistent or adapt across spatial transformations, independently of task demands^28^. Additionally, by defining task relevance through explicit instructions to detect certain stimuli as targets, the paradigm enables a systematic investigation of how task goals influence neural responses, allowing the dissociation of task-related processing from neural activations associated with visual perception^29^.

This paper focuses solely on the iEEG data, including behavioral, eye tracking, iEEG recordings, and rich metadata specifications. The primary goal of sharing this dataset is to provide researchers with a meticulously curated repository of standardized high-quality iEEG recordings collected from three academic medical centers with the same experimental protocol ensuring generalization across populations, recording systems, experimenters, and patient populations.

**Fig. 1:**
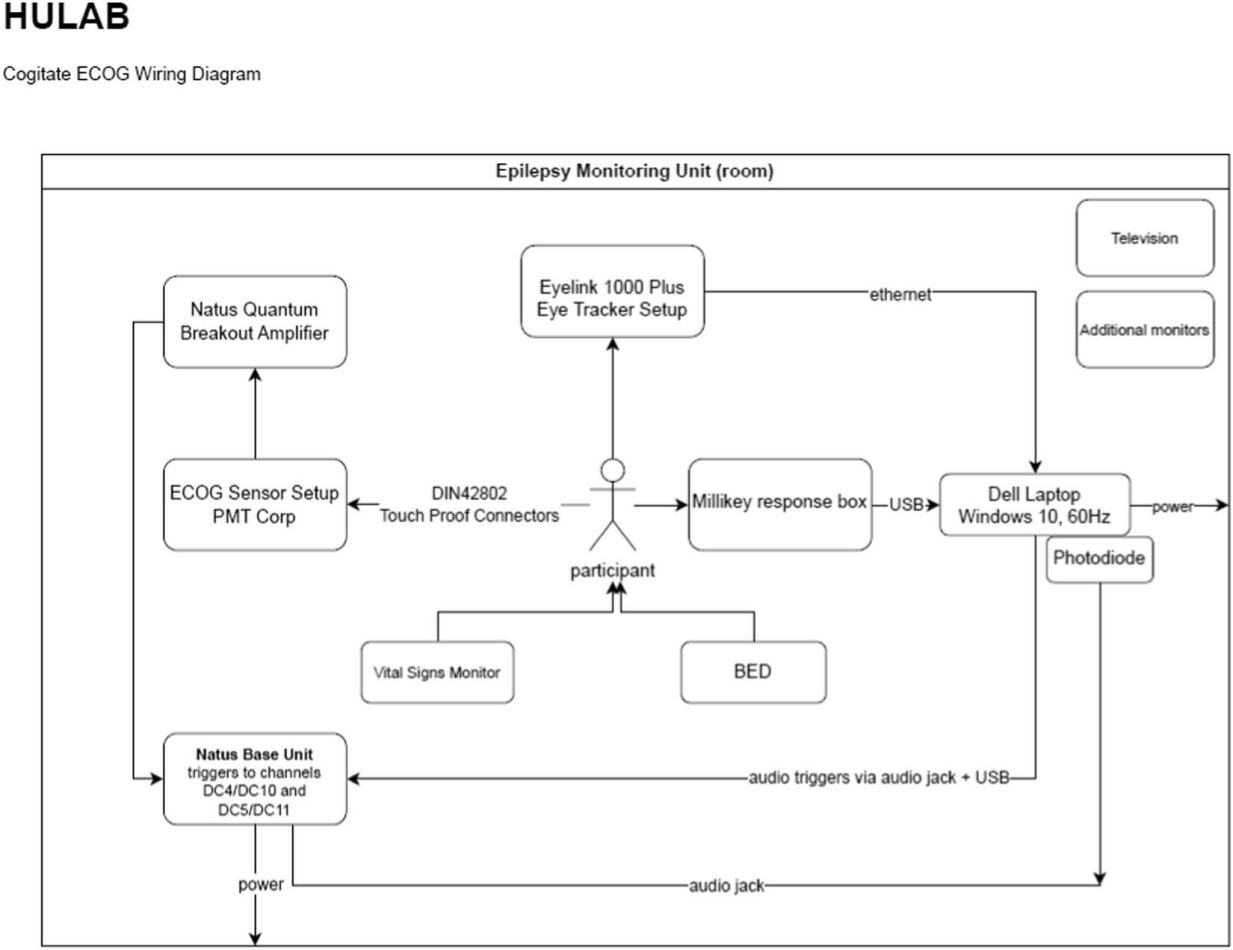
Experimental setup from one of the recording sites. Intracranial EEG (iEEG) data were transmitted from electrodes in the head, using touch-proof connectors to the data amplifier, and then to the base unit. Simultaneously, eye-tracking data, and behavioral data were collected via an eye-tracker and response box, respectively. The experimental PC sent photodiode pulses through the amplifier to the base unit. Extra efforts were made to standardize the acquisition setups and experimental context across the three laboratories. To that end, the same experimental setup was used across three laboratories, albeit with different amplifiers and eye-trackers. A shared standard operating procedure enabled comparable acquisition conditions across experimenters and labs. The above diagram corresponds to the experimental setup at Boston Children’s Hospital and Brigham and Women’s Hospital (Harvard Medical School). To ensure participants remained isolated from power sources, all computer systems (including eye-tracking equipment) were powered via an isolator box (NYU), an isolated ground outlet (WU), or a battery (HU). Wiring diagrams and further details for each laboratory are summarized in supplementary figures.

The dataset also includes metadata going beyond the minimum standards established by BIDS by incorporating extensive details ranging from data collection specification to clinical information, as recommended by Mercier *et al*.^3^ and Zheng *et al*.^30^. This effort represents a strong foundational commitment to fostering scientific inquiry and advancing the frontiers of our understanding of the human brain. We aim to empower our peers with this robust collection, and hope it supports future innovative studies that enable the scientific community to delve deeper into the neural basis of visual cognition (Fig. 1).

## Methods

### Participants

Data from 38 pharmaco-resistant epilepsy patients are shared. The dataset includes: iEEG recordings, vital signs (EKG, for a subset of patients, see supplement Table 1), eye-tracking data, behavioral data, wiring diagrams for equipment used at each site (summarized in Fig. 1 and in supplement Fig. 1), standard operating procedures (SOPs) for data collection, and clinical background information for every patient. They were all collected at the Comprehensive Epilepsy Center at New York University (NYU) Langone Health, Brigham and Women’s Hospital, Boston Children’s Hospital (Harvard Medical School), and University of Wisconsin School of Medicine and Public Health (WU).

The experiment and associated data collection devices were reviewed and approved by each of the site’s independent institutional ethics committees (NYU Langone Health Institutional Review Board (IRB), Boston Children’s Hospital IRB, Brigham and Women’s Hospital IRB, and University of Wisconsin-Madison IRB). Before obtaining consent, all participants were confirmed to have the cognitive capacity to provide informed consent by a member of the patient’s clinical care team. Upon receiving confirmation of cognitive capacity, all participants provided oral and written informed consent before beginning study procedures. They were informed that participation was strictly voluntary, and would not impact their clinical care. Participants were informed that they were free to withdraw participation in the study at any time, and that their data would be shared publicly following de-identification protocols. When recruiting minors, assent was obtained from minors and informed consent for data collection and data sharing was obtained from a parent or legal guardian. All study procedures were conducted in accordance with the Declaration of Helsinki (Table 1).

#### Inclusion criteria

We recruited 38 participants (23 female) between the ages of 10 and 65 years (M = 29.89, SD = 13.06). Adults provided both written and oral informed consent, while children provided written assent, accompanied by written and oral informed consent from a parent or legal guardian. Participants had an IQ of >70, with self-reported normal hearing, normal or corrected-to-normal vision, and cognitive and language abilities within or above the normal range in formal neuropsychological testing performed before surgery, when available. The study team postponed testing in cases where participants experienced an electrographic seizure within 3 hours of scheduled testing, until the patient was comfortable proceeding with the experiment, and no clinical contraindications to completing the experiment were identified. When available, information concerning language dominance as assessed by the intracarotid sodium amobarbital (WADA^31,32^) is reported (see Table S1). Given the clinical context of the study, participants completed testing at various points during their surgical admission and therefore may have been on a range of medications including, but not limited to, steroids, antibiotics, pain relievers, or medications used to treat unrelated conditions. While anti-seizure medications are typically titrated down during the monitoring period, this process occurs gradually over several days, meaning the exact medication status and dosage varies across participants.

#### Exclusion criteria

Participants who were unable to complete a sufficient number of trials due to excessive muscular artifacts, movement, noisy recordings, or a decision by the participant to terminate the experiment were excluded. Out of the 42 recorded datasets, 38 met the inclusion criteria and were included in the shared sample, while 4 were excluded (one participant had only a single block recorded, another contained corrupted data, and two were recorded without triggers).

### Experimental design

**Fig. 2:**
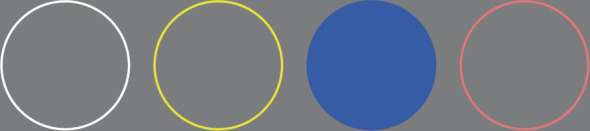
Schematic of the Finger Localizer task. During each trial, participants viewed four outlined circles (white, yellow, blue, and pink) arranged in a row. One circle filled with its corresponding color, signaling participants to press the matching colored button on the response box as quickly as possible. In this example, the blue circle is filled, indicating the correct response on the response box would be the blue button.

IEEG data were collected on two tasks: a finger localizer task, and a Go/No-Go detection task. First, participants took part in a finger localizer task, which was aimed at identifying relevant motor areas associated with finger movements, particularly those motor responses executed during the Go/No-Go task. This control task ensures that neural responses associated with motor responses in the Go/No-Go task are correctly localized. During this task, participants were presented with four circles outlined in different colors matching the colors of the buttons on the response box (Fig. 2). Arranged in a row from left to right, these colors were: white, yellow, blue, and pink. In each trial, a different circle would fill with the color of its outline, signaling them to press the corresponding colored button on the response box as soon as possible. The colored buttons on the response box were arranged in the same order as the circles presented on the laptop screen during the task. The filled circle persisted throughout the duration of the response period, followed by an additional delay of 200 milliseconds. Inter-Trial Intervals (ITIs) followed a uniform distribution, averaging 0.55 seconds and ranging from 0.40 to 0.70 seconds. This experiment consisted of 80 trials, evenly distributed across the four colors (20 trials per color), in a randomized sequence.

**Fig. 3:**
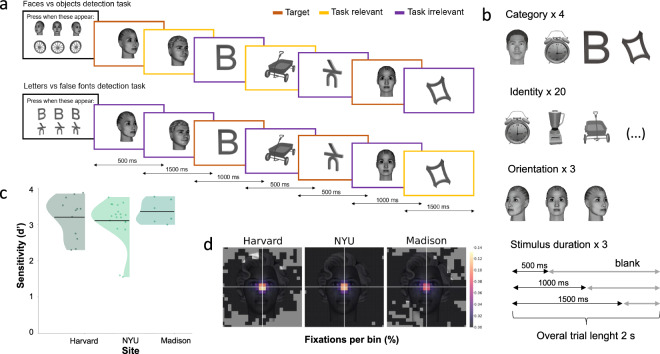
Experimental design and datasets summary. (**a**) Overview of the experimental paradigm, showing two example blocks of trials: At any moment, no more than one high-contrast stimulus was present at fixation. In each trial, participants were asked to detect target stimuli: either a face and an object or a letter and a false font in any of the three different orientations. Thus, each trial contained three stimuli types: targets (depicted in orange), task-relevant stimuli (belonging to the same categories as the targets, depicted in yellow), and task-irrelevant stimuli (belonging to the two other categories, depicted in purple). Colored frames are used here for illustration purposes only and did not appear in the experiment. The pictorial stimuli (faces/objects) were task-relevant in half of the blocks (upper row), while the symbolic stimuli (letters/false fonts) were relevant in the other half of the blocks (lower row), and vice versa. Blank intervals between stimuli were also included but are not depicted here. (**b**) The stimulus properties we manipulated were category (objects, faces, letters and false fonts), identity (each category contained 20 different exemplars), orientation (left, right, and front view), and duration (0.5, 1.0, and 1.5 seconds). Example stimuli used in the study are shown here; for the full stimulus set see here. (**c**) Distribution of behavioral sensitivity scores (d’) separate for each of the three data acquisition sites. Horizontal black lines depict average d’ per site, and dots depict individual participants’ d’s. Average accuracy: M = 95.52 (SD = 7.58). Average reaction time (RT): M = 0.64 s (SD = 0.14). (**d**) Average fixation (Eyelink)/gaze coordinates (Tobii) heat maps computed over a 0.5 s window after stimulus onset, zoomed into the stimulus area for each recording site.

In the main experiment, participants performed a visual Go/No-Go matching task with five critical experimental manipulations implemented in a factorial design. Stimuli were (1) of different categories (faces, objects, letters, and false-fonts), (2) identities (20 per category), and were presented (3) in different orientations (front, left and right views) and (4) for different durations (0.5, 1.0, and 1.5 sec, see Fig. 3b). The fifth factor, task relevance, had three levels: targets (which participants had to remember and press a button when appearing on the screen), task-relevant non-targets (of the same category as the target stimuli, but of a different identity) and task-irrelevant stimuli (of a different category than the targets, see Fig. 3a).

### Stimuli

Stimuli covered approximately 6 × 6° of visual angle area on the screen. Faces were created with FaceGen Modeler 3.1; letter and false font stimuli were generated with MAXON CINEMA 4D Studio (RC - R20) 20.059; object stimuli were selected from the Object Databank^33^. All stimuli were gray-scaled and scaled for equal luminance and size using the SHINE toolbox^34^. To aid target identification, faces varied in hairstyle, ethnicity and gender. Stimulus orientation was balanced such that half of each category had a side view (equally facing either left by 30° or right by −30°). The remaining half were front views.

### Experimental procedure

The Go/No-Go task was divided into 20 blocks, with breaks between blocks paced by the participant. At the beginning of each block, two target stimuli were presented: a face and an object or a letter and a false font. Participants were instructed to press a button whenever they detected the occurrence of a target stimulus (face/object or letter/false font). Targets did not repeat across blocks. The blocks were ordered in an AABB sequence, where two consecutive face-object blocks were always followed by two consecutive letter-false font blocks and the pairing order was counterbalanced across the experiment.

All stimuli were presented foveally for one of three durations (0.5, 1.0, and 1.5 seconds). Next, a blank screen was presented to extend each trial to 2.0 sec, followed by an additional jitter lasting for an average duration of 0.4 s (truncated exponential distribution between 0.2 and 2.0 s, median absolute deviation≈0.1 s). This resulted in a mean trial length of 2.4 s. Participants were instructed to maintain focus on a central fixation cross in between trials.

Within each mini-block, half of the stimuli were task-relevant (i.e., they belonged to the same faces-object or letters-false fonts categories as the targets) and half task-irrelevant (i.e., they were from the two other categories). The stimulus identity varied randomly while appearing equally across trial durations and task conditions (Fig. 3).

### Data collection harmonization

To minimize discrepancies between data collection sites, experimental devices were standardized across sites to the extent possible. The same response box (Millikey LH-8) was used across sites. For HU and NYU, the same laptop was used (Dell Precision 5540 laptop, with a 15.6” Ultrasharp screen), while a Dell D29M PC with an Acer 19.1” screen was used in WU. Across all three sites, we used the same custom photodiode device, positioned at a corner of the display to record luminance changes in a small square that switched from black to white. This captured the exact frame of each stimulus onset and offset. The photodiode signals were recorded alongside the iEEG channels, enabling offline extraction of event onsets to synchronize the recorded signals with on-screen events presentation. Except for three participants at WU (whose amplifier defaulted to a TTL output), the photodiode signal was recorded as an analog input. An audio/USB trigger system was available as a backup. Before data collection, a procedure similar to that described in Lepauvre *et al*.^35^ was applied in each data acquisition site to ensure consistent timing and experimental design across sites.

At WU, clinical constraints prevented recording the photodiode on the same amplifier as the iEEG channels, as such the photodiode was recorded on a separate amplifier (Blackrock for the first three datasets, analog; Neuralynx for subsequent datasets, TTL). Because separate amplifiers run on different clocks, a subset of iEEG channels was duplicated onto those amplifiers, allowing the two recordings to be aligned via cross-correlation. This additional step precisely synchronized the photodiode signal with the main iEEG recordings (see technical validation).

To mitigate potential procedural differences across sites, we developed a standardized operating procedure (SOP) outlining instructions for conducting experiments and which is shared alongside the data (https://cogitate-consortium.github.io/cogitate-data/08_links/#links-and-reference-materials, iEEG SOP). The SOP encompassed participant instructions, setup guidelines, and general experiment protocols. Although the instructions to participants and the way the task was conducted remained consistent across sites, the SOP also accommodated logistical differences specific to each location—such as the source of necessary equipment, how to configure power connections (e.g., using a battery at Harvard versus an isolated socket at WU), and the processes for data storage and retrieval after each session. This comprehensive approach to quality assurance not only involved technical considerations but also extended to the establishment of standardized procedures, ensuring that the data collected across diverse sites could be reliably compared and utilized for meaningful scientific investigations.

### Data acquisition

#### Behavioral data acquisition

The task was run on Matlab (The MathWorks Inc., 2019, Harvard: R2020b; NYU: R2020a, WU: 2021a)^36^ using Psychtoolbox v.3.4^37^. NYU and Harvard used a Dell Precision 5540 laptop, with a 15.6″ screen and WU used a Dell D29M PC with an Acer 19.1″ V196WL screen. Participants responded using an 8-button response box (Millikey LH-8; response hand(s) varied) using the button of their choice for targets.

#### Eye tracking data acquisition

Eye tracking and pupillometry data were collected using an EyeLink 1000 Plus on remote mode, sampled monocularly at 500 Hz (from the left eye at WU, and from the left or right eye depending on the setup at Harvard), or on a Tobii-4C eye-tracker, sampled binocularly at 90 Hz (NYU, see Table 2). Eye trackers were calibrated at the beginning of the task (using a 13 points calibration for HU and WU, 9 points for NYU), and could be recalibrated as needed before every fourth block. For the eyelink eye trackers, the eyetracking recordings were synchronized with the behavioral tasks using triggers, which were sent via an ethernet protocol from the experimental computer to the Eyelink computer to mark each critical event. In the case of the Tobii eye-tracker, as the data were recorded directly on the experimental computer, no further synchronization was required.

**Table 1:** iEEG patients demographics.

Participant ID	Gender	Age	Handedness	Electrode Scheme	# electrodes	Hemispheric electrode placement
CE103	F	49	left	sEEG	58	bilateral
CE106	F	18	right	sEEG	118	left
CE107	M	24	right	sEEG	168	left
CE108	F	16	right	sEEG	108	left
CE109	F	50	right	sEEG	104	bilateral
CE110	F	15	right	sEEG	186	right
CE112	F	17	right	sEEG	158	right
CE113	F	26	ambidextrous	sEEG	60	bilateral
CE115	M	17	right	sEEG	88	right
CE118	M	11	right	sEEG	164	left
CE119	M	29	right	sEEG	104	bilateral
CE120	M	12	left	sEEG	164	left
CE121	M	20	right	sEEG	191	left
CF102	F	30	right	ECoG & sEEG	133	left
CF103	M	24	right	ECoG & sEEG	189	left
CF104	F	23	right	ECoG & sEEG	116	left
CF105	M	31	right	ECoG & sEEG	176	left
CF106	M	17	right	ECoG & sEEG	156	left
CF107	F	31	right	ECoG & sEEG	242	right
CF109	F	30	left	ECoG & sEEG	102	right
CF110	F	17	right	ECoG & sEEG	174	left
CF112	M	23	right	ECoG & sEEG	180	bilateral
CF113	F	38	right	ECoG & sEEG	132	right
CF116	M	43	left	sEEG	166	bilateral
CF117	M	28	right	sEEG	174	bilateral
CF119	M	37	right	ECoG & sEEG	104	left
CF120	F	61	right	sEEG	79	left
CF121	F	50	right	sEEG	75	right
CF122	F	27	right	sEEG	104	right
CF124	F	33	right	sEEG	146	bilateral
CF125	F	23	right	sEEG	138	left
CF126	F	23	right	sEEG	122	left
CG101	M	40	right	sEEG	104	bilateral
CG102	M	49	right	sEEG	86	bilateral
CG103	F	57	right	sEEG	76	bilateral
CG104	F	48	right	sEEG	72	bilateral
CG105	F	24	right	sEEG	70	bilateral
CG106	F	25	ambidextrous	sEEG	76	bilateral

Abbreviations: (F) female, (M) male, (ECoG) electro-corticography, (sEEG) stereo electro-encephalography. CE, CF, and CG denote the medical center in which the data were acquired.

#### IEEG data acquisition

Intracranial brain activity was recorded using varying combinations of the following platinum-iridium electrodes depending on the recording site: subdural grids embedded SILASTIC sheets, or depth stereo-electroencephalographic, or Ad-Tech macro-micro depth electrodes (3 to 5.5 mm spacing, micro wires data were not collected for the shared tasks). Grids had 8 × 8 contacts with 10 mm center-to-center spacing, 8 × 16 contacts with 3 mm spacing; or hybrid macro/micro 8 × 8 contacts with 10 mm inter-contact distance, and 64 embedded microcontacts with 5 mm inter-contact distance. Linear strips had 4–12 contacts with 10 mm inter-contact distance, and depth electrodes had 8 to 12 contacts with 1.5 to 2.43 mm inter-contact distance. Macro grids, micro grids, strips and stereo EEG electrodes were acquired using 256-channels NATUS amplifier systems across all recording sites (Table 2), with a sampling frequency varying between 512 and 2048 Hz across participants, and are shared at their original sampling rate. The data includes a total of 4771 electrodes across 38 participants (1238 surface, 3533 depths).

#### Electrodes reconstruction

**Fig. 4:**
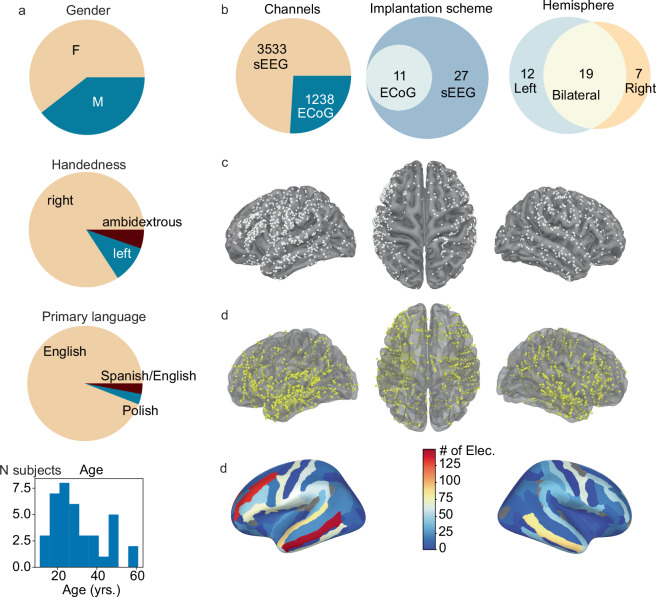
Summary of electrodes coverage. (**a**) Participants’ demographic information including summary reports of distribution of (self-reported) gender, handedness, primary language and age across participants. (**b**) Summary of electrode counts and implantation schemes. The left pie chart depicts the total number of cortical (ECoG) and stereo-electrodes (sEEG) in the data set. The middle Venn diagram (implants) represents the number of participants implanted with only sEEG or both ECoG and sEEG. The right Venn diagram (scheme) represents the number of participants with electrodes located in the left hemisphere, right hemisphere or both hemispheres. (**c**) ECoG channels localization are depicted in white on fsaverage template brain. (**d**) sEEG channels localization are depicted in yellow on fsaverage template brain. (**e**) Number of electrodes in each region of interest based on the Destrieux cortical parcellation (74 labels per hemisphere).

Across all sites, post-implant computed tomography (CT) images were co-registered with the pre-implant T1 MRI images using FLIRT^38^ as implemented in FSL^39^. Individual pial surfaces were reconstructed based on T1 MRI using the Freesurfer image analysis suite (‘recon-all’). Electrode T1 coordinates were obtained by localizing the electrodes on the CT scan using site-specific custom algorithms and pipelines that reflect each site’s standard practices. For NYU participants, electrode labels were assigned semi-automatically or manually using FLSView^39,40^. For surface electrode grids, three corner electrodes were localized manually and the remaining electrode coordinates were then automatically interpolated along the shared plane using the known inter-electrode distances. A custom algorithm estimates the surface under each grid along the curve of a sphere larger than the brain. The algorithm then iteratively adjusted the projection of the grid plane, minimizing the error between the projected and known electrode locations. Electrode locations were then adjusted for estimated brain shift/swelling^41^. Subdural strips were localized manually. If sEEG depths did not follow a straight trajectory, they are localized manually. For WU participants (sEEG only), electrode labels were assigned manually or semi-manually using the SubNuclear toolbox (https://github.com/ckovach/SubNuclear). For Harvard participants (sEEG only), individual depth electrode contacts were labelled manually from CT using BioImageSuite’s Electrode Editor tool^42^, and converted to coordinates within T1 MRI-space with the iELVis toolbox^41,43^. For all sites, electrodes were converted from subject-specific T1 space to a common MNI space using either surface-based (cortical surface electrodes) or a linear-based transformation (depths) using the freesurfer average brain (fsaverage, MNI305^44^, Fig. 4) (Table 2).

**Table 2:** List of devices used to record iEEG data, eye-tracker (ET) data, structural MR scans (MR) and CT data across the different sites.

	iEEG	ET	MR (T1 pre-op)	CT (post-op with electrodes)
HU	NATUS Amplifier System	Eyelink 1000 Plus	n/a*	n/a*
NYU	NATUS Amplifier System	Tobii Eye tracker (IS4LPOO1)	SIEMENS 3 T Biograph_mMR	SIEMENS SOMATOM Force
WU	NATUS Amplifier System	Eyelink 1000 Plus	GE MEDICAL SYSTEMS 1.5 T (Optima Artist, Optima MR450w), 3 T (Optima Architect)	Canon Medical Systems Aquilion ONE

*MRI and CT scans from Harvard University are not included in the data release as those could not be openly shared.

## Data Records

### Data release formats/naming conventions

The raw and BIDS (Brain Imaging Data Structure) formats of the data are available in two ways: (1) Archival Format (Bundles in a zip format on our website https://www.arc-cogitate.com/data-release^45,46^), and (2) XNAT (eXtensible Neuroimaging Archive Toolkit at http://cogitate-data.ae.mpg.de/^47^). We chose to make the data available in two different ways to cater to various users and their various degrees of proficiency with digital tools.

#### Data bundles

##### Raw format

**Fig. 5:**
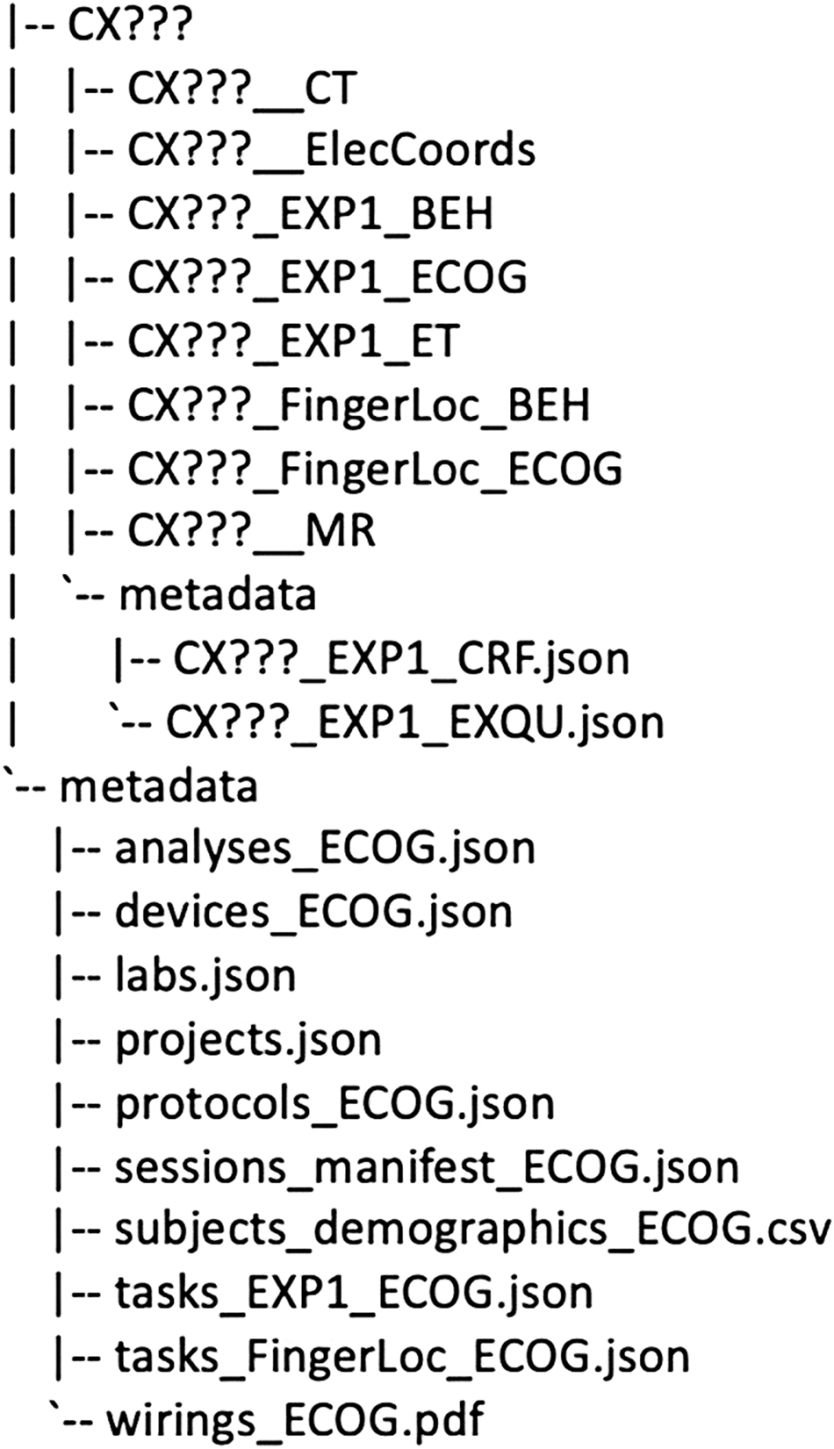
Raw data bundles folder structure.

Raw data bundles^45^ follow the below naming convention. The project root is organized in sub-folders, where each participant’s data is stored in a dedicated folder. The participant code is in the format “CX???”, where the two first letters reflect the site where the data were collected and the question marks represent the participant ID. The participant directories consist of various sub-directories along with a metadata folder that contains CRF (Case Report Form, containing notes taken by the experimenter during the recordings) and EXQU (Exit Questionnaire, containing the participants’ responses to the feedback questionnaire provided at the end of the experiment). Each subfolder follows the naming pattern SUBJECT_PARADIGM_MODALITY. SUBJECT refers to the participant ID. PARADIGM refers to the experimental paradigm used to collect the data, and MODALITY refers to the data type stored within the folder. For each participant, we recorded CT scans (CT), MR scans (MR), behavioral data (i.e., participants’ responses, BEH), eye-tracking data (ET), and iEEG data (ECOG) from the experimental task. Additionally, we collected event-related behavioral data (FingerLoc_BEH) and iEEG data (FingerLoc_ECOG) during the Finger Localization task. The dataset also includes files related to electrode coordinates, labels, and other metadata containing essential information about events and channels, all of which can be found in the ElecCoords directory. The CT and MR scans were acquired to obtain anatomical data of the participant’s brain and to localize iEEG electrodes; no experimental paradigm was used for these scans. The MR and CT data are shared in their native, DICOM format. The PARADIGM is therefore left empty for MR and CT scans. In addition, the root directory also contains a metadata folder that includes several key files: a link to the analysis code (analysis_ECOG.json), a list of devices used for data acquisition (devices_ECOG.json), information on the labs involved in data collection along with other relevant details (labs.json and projects.json), a manifest of MR and CT datasets (sessions_manifest_ECOG.json), the experimental protocol for the task (protocols_ECOG.json), participants’ demographic data (subjects_demographics_ECOG.json), descriptions of the experimental paradigm (tasks_EXP1_ECOG.json), details of the Finger Localizer task (tasks_FingerLoc_ECOG.json), and a wiring diagram showing how the devices were connected (wirings_ECOG.pdf). Figure 5 illustrates the directory structure for raw data and the format for each type of data is detailed in Table 3.

**Table 3:** Naming conventions and data formats.

Data type	Folder	File naming conventions	Data formats
CT scan	CX???__CT	CX???_CT_1.nii.gz or CX???__CT/*.dcm	DICOM or NIFTI
Behavioral data	CX???_EXP1_BEH	CX???_Beh_1_RawDurR?	CSV
iEEG data	CX???_EXP1_ECOG	CX???_ECoG_1_DurR?	EDF
Eye-tracking data	CX???_EXP1_ET	CX???_ET_1_DurR?	ASC or CSV
MR scan	CX???__MR	CX???_MR_1.nii.gz or CX???__MR/*.dcm	DICOM or NIFTI
iEEG data for the Finger Localizer task	CX???_FingerLoc_ECOG	CX???_ECOG_1_FingerLoc	EDF
Behavioral data for the Finger Localizer task	CX???_FingerLoc_BEH	CX???__FingersLocalizer_LOG	CSV
Electrode coordinates and additional data files	CX???_ElecCoords	CX???_ses-1_atlas-desikan_labels CX???_ses-1_atlas-destrieux_labels CX???_ses-1_laplace_mapping_ieeg CX???_ses-1_space-fsaverage_electrodes CX???_ses-1_space-fsaverage_coordsystem CX???_ses-1_task-Dur_channels CX???_ses-1_task-Dur_events	TSV and JSON

##### BIDS data

**Fig. 6:**
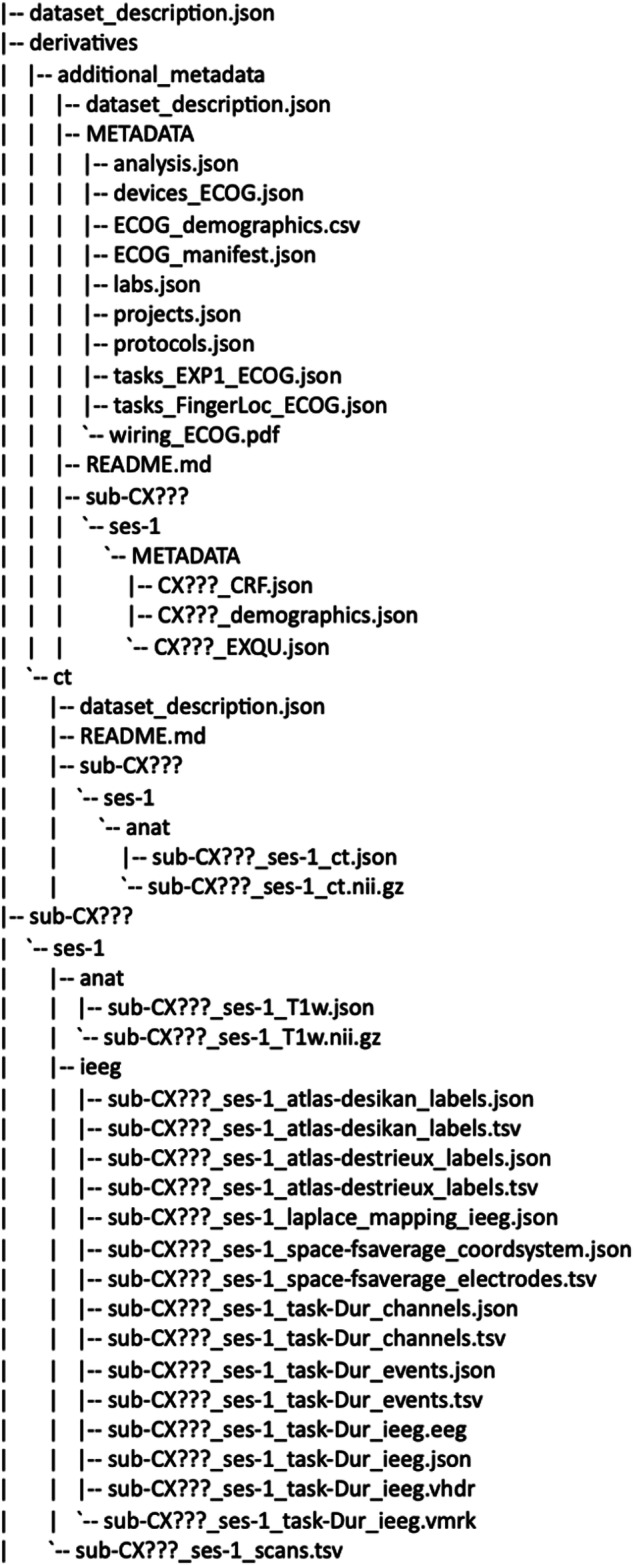
BIDS data bundles folder structure.

The raw data were also converted to BIDS using the MNE-bids package^48^. The BIDS root^46^ (Fig. 6) contains a subfolder for each participant, using the format “sub-CX???”. The participant’s folder contains a nested folder structure, with the highest level referring to the session the recording was collected from (ses-1), followed by the main iEEG data folder (ieeg) and MR scans data folder (anat). The data files are located at the lowest level of the directory structure (see Table 4). The iEEG data are stored in the BrainVision format named sub-CX???_ses-1_task-PARADIGM_ieeg. The events associated with the experimental task are stored in a tsv file format under the name sub-CX???_ses-1_task-PARADIGM_events.tsv and are accompanied by a json file of the same name containing information about the events. Electrode localization is stored in the tsv file sub-CX???_ses-1_space-fsaverage_electrodes.tsv in MNI space (fsaverage), as described in the associated sub-CX???_ses-1_space-fsaverage_coordsystem.json file (see electrodes reconstruction section). In addition, the sub-CX???_ses-1_task-Dur_channels.tsv file contains additional information about each channel in the recording, such as the type of electrode (seeg or ecog), online filtering used during data collection, and sampling frequency. In addition, we provide a status description for each electrode, containing annotations about the epileptic activity characterized by an epileptologist (only available for NYU data, participants CF102-CF126 in Table 1) and about noise levels based on visual inspection. For further specification, consult the BIDS specification for iEEG^11^. Finally, the Laplace mapping json files contain the scheme to be used for Laplace re-referencing, subtracting activation of neighboring electrodes of a given electrode. Additional metadata (case report forms, exit questionnaires, subject demographics, information related to the experimental setup etc.) and CT scans are included along with the BIDS data under derivatives/additional_metadata and derivatives/ct respectively (Fig. 6 and Table 4)

**Table 4:** Naming conventions of the BIDS converted iEEG data.

Data type	File naming conventions	Data formats
Experimental events	sub-CX???_ses-1_task-Dur_events	TSV and JSON
iEEG data	sub-CX???_ses-1_task-Dur_ieeg	BrainVision (vmrk, vhdr, eeg)
Electrodes coordinates	sub-CX???_ses-1_space-fsaverage_electrodes sub-CX???_ses-1_space-fsaverage_coordsystem	TSV and JSON
Channels information	sub-CX???_ses-1_task-Dur_channels	TSV and JSON
Laplace mapping	sub-CX???_ses-1_laplace_mapping_ieeg	JSON
CT scan	sub-CX???_ses-1_ct	NIFTI and JSON
MR scan	sub-CX???_ses-1_T1w	NIFTI and JSON
Electrode labels	sub-CX???_ses-1_atlas_destrieux_labels subCX???_ses-1_atlas_desikan_labels	TSV

#### XNAT

##### Raw format

To simplify data downloading and offer more flexibility for accessing specific data, the raw data is also available on the XNAT platform^47^. A key advantage of the XNAT release is that it enables searches over the data based on predefined criteria (e.g., gender, age, etc) in addition to the separate download of the data based on those predefined criteria. While the data content is identical to that of the raw bundles (which only allows for download of the complete dataset), it is organized in a different structure. The raw data is arranged as follows: In the project directory, a list of participants along with demographic information is available. Under the ‘Resources’ section, project-level metadata such as demographics (for all participants), devices, analyses, protocols, and more can be found. Within each subject folder, EyeTracker and iEEG data are located under ‘Experiments’. Subject-specific demographics are available under the subject’s ‘Resources’. Inside the iEEG experiment folder, data files such as BEH and BEHFingerLoc are stored under ‘Resources’. Additionally, metadata files, including CRF and EXQU, are present in this directory. MR and CT scans are available in two formats: NIFTI and DICOM. NIFTI scans can be found within the directory, while DICOM scans are organized as separate sessions, alongside ECOG and ET data, under ‘Experiments’.

##### BIDS converted raw data

The BIDS format of the data follows a similar structure and file organization as the BIDS data bundles.

## Technical Validation

Data were collected by three independent laboratories to ensure generalization across patient populations, recording systems, and experimenters. The exact timing of the stimulus presentation was recorded with the photodiode. Response boxes used by all laboratories have reported an average of one-millisecond latency (Millikey, LabHakkers).

The data were checked at three levels (based on the principles described in Gorska-Klimowska*, Hirschhorn* *et. al*., in preparation). Firstly, we ensured that the data contained all expected files (see Table 1), that they were of consistent naming conventions (see Table 3), and that all personal information had been removed. Personal information was removed through on-site programmatic editing of the files headers. Additionally, programmatic verification was implemented on our shared data repository (XNAT) to reject any data with improper anonymization. Secondly, we established that our task manipulations were effective by testing behavior and eye-tracking data. This revealed that patient performance was relatively high (hit rate 94.89%, SD = 4.22, false alarm rate 2.39% SD = 2.02; Fig. 3c), with only three participants having hit rates <70% and false alarm rates >30%. Moreover, fixations remained relatively stable for all the participants throughout the entire experiment duration (Fig. 3d). Finally, we checked the quality of the neural data. Channels localized within the epileptic onset zone were marked at NYU and WU by a certified epileptologist, and two independent validators marked contacts that were either damaged (flat or noisy) or implanted outside the brain tissue. This information was stored in the sub-CE103_ses-1_task-Dur_channels.tsv files. Overall noise in the data was assessed by computing the summary spectra before and after notch filtering. Participants’ data were excluded in cases of technical issues during recording (missing photodiode triggers or corrupted data preventing proper file reading) or if they completed only a single block, which was insufficient for analysis. As a result, four datasets were excluded from the data release (three from NYU, one from WU).

Localization of the electrode contacts was validated by multiple steps of visual inspection by two independent validators. The location of each electrode contact was checked with respect to the individual anatomy. Individual labels were checked for their accordance with atlases, and atlas mapping in BIDS was further compared with the initial atlas outputs. Finally, to verify alignment, we plotted the time intervals between consecutive triggers in the neural recordings against the intervals between corresponding events in the log file, ensuring a precise match. Notably, the WU site required a cross-correlation procedure (using variable-sized kernels and detecting peaks above the mean correlation SD; see code for details) due to triggers being recorded on non-clinical amplifiers (see Data collection harmonization section above). The data being shared contains data where triggers have been aligned and further verified with the tests above. Pre-alignment data can be available upon request.

## Usage Notes

The data can be accessed via our live XNAT database (http://cogitate-data.ae.mpg.de/^47^) which offers a web interface to navigate through the existing data and selectively download specific data (of specific participants, sessions, etc.). An API is also available to download the data programmatically (https://wiki.xnat.org/documentation/the-xnat-api). Alternatively, data bundles can be downloaded from our website in a zip format (https://www.arc-cogitate.com/data-release^45,46^). In both cases, users must create an account before gaining access to the downloading interfaces. More detailed information is available on our documentation website (https://cogitate-consortium.github.io/cogitate-data/).

We further provide code to preprocess the data, identify onset responsive channels and perform temporal decoding of categorical information (e.g., faces vs. objects), illustrated in a Jupyter notebook (notebooks/ieeg-data-release.ipynb). Additionally, pipeline scripts with default parameters are available for analyses on all channels and participants. All provided pipelines are fully customizable through JSON files (docs/config-default.json).

The preprocessing pipeline, based on Cogitate *et al*.^13^, includes the removal of line noise (60 Hz), identifying and excluding defective channels based on visual inspection and clinical annotations, re-referencing using a Laplace scheme, computing high gamma power (70–150 Hz) and event-related potentials (ERPs, 0–30 Hz), as well as epoching around stimuli onsets and offsets. Standard iEEG analysis pipelines are provided for identifying onset-responsive channels and decoding categorical information (e.g., faces vs. objects). For instance, onset response detection compares high gamma activation pre- (−0.3 to 0 sec) and post-stimulus (0.05 to 0.350 sec), using a paired t-test. Cross-temporal generalization^49,50^ for decoding faces vs. objects employs support vector machines and 5-fold cross-validation. We also demonstrate how anatomical electrode labels from Freesurfer reconstruction atlases can be used to constrain analyses spatially, enabling investigating neural dynamics in specific brain regions. Finally, a Jupyter notebook (ieeg-single-participant-report.ipynb) generates a comprehensive report for each participant, preprocessing data and computing onset responsiveness, with results displayed on the fsaverage brain.

## Supplementary information

Supplementary information

## Data Availability

The Matlab code used to run the experiment is available at https://github.com/Cogitate-consortium/cogitate-experiment-code. The code implementing the preprocessing pipeline and analysis scripts is accessible at https://github.com/Cogitate-consortium/iEEG-data-release^51^. All code is implemented in Python using MNE-python v.1.7^52^ and Matlab. The repository’s README file provides an overview of the codebase and instructions on how to set up the environment. While the README offers an overall guide, we recommend that users consult the Jupyter notebook (ieeg-data-release.html) available in the repository for detailed usage instructions. This notebook showcases how to download the data from our repository, implement the described analyses, and provides additional information on interacting with the dataset, including selecting specific conditions and customization options. Extensive information about the experimental paradigm, recording modalities and more can be found on the accompanying wiki: https://cogitate-consortium.github.io/cogitate-data/.

## References

[1] Ryvlin, P., Cross, J. H. & Rheims, S. Epilepsy surgery in children and adults. *Lancet Neurol.* **13**, 1114–1126, 10.1016/S1474-4422(14)70156-5 (2014). 25316018 10.1016/S1474-4422(14)70156-5

[2] Jobst, B. C. *et al*. Intracranial EEG in the 21st Century. *Epilepsy Curr.* **20**, 180–188, 10.1177/1535759720934852 (2020). 32677484 10.1177/1535759720934852 PMC7427159

[3] Mercier, M. R. *et al*. Advances in human intracranial electroencephalography research, guidelines and good practices. *NeuroImage* **260**, 119438, 10.1016/j.neuroimage.2022.119438 (2022). 35792291 10.1016/j.neuroimage.2022.119438 PMC10190110

[4] Jacobs, J. & Kahana, M. J. Direct brain recordings fuel advances in cognitive electrophysiology. *Trends Cogn. Sci.* **14**, 162–171, 10.1016/j.tics.2010.01.005 (2010). 20189441 10.1016/j.tics.2010.01.005 PMC2847661

[5] Johnson, E. L., Kam, J. W. Y., Tzovara, A. & Knight, R. T. Insights into human cognition from intracranial EEG: A review of audition, memory, internal cognition, and causality. *J. Neural Eng.* **17**, 051001, 10.1088/1741-2552/abb7a5 (2020). 32916678 10.1088/1741-2552/abb7a5 PMC7731730

[6] Lachaux, J.-P., Axmacher, N., Mormann, F., Halgren, E. & Crone, N. E. High-frequency neural activity and human cognition: Past, present and possible future of intracranial EEG research. *Prog. Neurobiol.* **98**, 279–301, 10.1016/j.pneurobio.2012.06.008 (2012). 22750156 10.1016/j.pneurobio.2012.06.008 PMC3980670

[7] Liu, J. & Xue, G. What Is the Contribution of iEEG as Compared to Other Methods to Cognitive Neuroscience? in *Intracranial EEG: A Guide for Cognitive Neuroscientists* (ed. Axmacher, N.) 103–124. 10.1007/978-3-031-20910-9_8 (Springer International Publishing, Cham, 2023).

[8] Berezutskaya, J. *et al*. Open multimodal iEEG-fMRI dataset from naturalistic stimulation with a short audiovisual film. *Sci. Data* **9**, 91, 10.1038/s41597-022-01173-0 (2022). 35314718 10.1038/s41597-022-01173-0 PMC8938409

[9] Buzsáki, G., Anastassiou, C. A. & Koch, C. The origin of extracellular fields and currents — EEG, ECoG, LFP and spikes. *Nat. Rev. Neurosci.* **13**, 407–420, 10.1038/nrn3241 (2012). 22595786 10.1038/nrn3241 PMC4907333

[10] Wilkinson, M. D. *et al*. The FAIR Guiding Principles for scientific data management and stewardship. *Sci. Data* **3**, 160018, 10.1038/sdata.2016.18 (2016). 26978244 10.1038/sdata.2016.18 PMC4792175

[11] Holdgraf, C. *et al*. iEEG-BIDS, extending the Brain Imaging Data Structure specification to human intracranial electrophysiology. *Sci. Data* **6**, 102, 10.1038/s41597-019-0105-7 (2019). 31239438 10.1038/s41597-019-0105-7 PMC6592874

[12] Gorgolewski, K. J. *et al*. The brain imaging data structure, a format for organizing and describing outputs of neuroimaging experiments.* Sci. Data* **3**, 160044, 10.1038/sdata.2016.44 (2016). 10.1038/sdata.2016.44 PMC4978148 27326542

[13] Cogitate, C. *et al*. Adversarial testing of global neuronal workspace and integrated information theories of consciousness. *Nature*10.1038/s41586-025-08888-1 (2025). 10.1038/s41586-025-08888-1 PMC12137136 40307561

[14] Melloni, L. *et al*. An adversarial collaboration protocol for testing contrasting predictions of global neuronal workspace and integrated information theory. *PLOS ONE* **18**, e0268577, 10.1371/journal.pone.0268577 (2023). 10.1371/journal.pone.0268577 PMC9916582 36763595

[15] George, M. A. *et al*. Conscious processing and the global neuronal workspace hypothesis. *Neuron* **105.5**, 776-798 (2020). 10.1016/j.neuron.2020.01.026 PMC8770991 32135090

[16] Dehaene, S. & Naccache, L. Towards a cognitive neuroscience of consciousness: basic evidence and a workspace framework. *Cognition* **79**, 1–37, 10.1016/S0010-0277(00)00123-2 (2001). 10.1016/s0010-0277(00)00123-2 11164022

[17] Dehaene, S. & Changeux, J.-P. Experimental and Theoretical Approaches to Conscious Processing. *Neuron* **70**, 200–227, 10.1016/j.neuron.2011.03.018 (2011). 10.1016/j.neuron.2011.03.018 21521609

[18] Dehaene, S., Kerszberg, M. & Changeux, J.-P. A neuronal model of a global workspace in effortful cognitive tasks. *Proc. Natl. Acad. Sci*. **95**, 14529–14534, 10.1073/pnas.95.24.14529 (1998). 10.1073/pnas.95.24.14529 PMC24407 9826734

[19] Dehaene, S., Lau, H. & Kouider, S. What is consciousness, and could machines have it? *Science* **358**, 486–492, 10.1126/science.aan8871 (2017). 10.1126/science.aan8871 29074769

[20] Albantakis, L. *et al*. Integrated information theory (IIT) 4.0: Formulating the properties of phenomenal existence in physical terms. *PLOS Comput. Biol.* **19**, e1011465, 10.1371/journal.pcbi.1011465 (2023). 37847724 10.1371/journal.pcbi.1011465 PMC10581496

[21] Tononi, G. Integrated information theory of consciousness: an updated account. *Arch. Ital. Biol.* **150**, 56–90, 10.4449/aib.v149i5.1388 (2012). 23165867 10.4449/aib.v149i5.1388

[22] Tononi, G., Boly, M., Massimini, M. & Koch, C. Integrated information theory: from consciousness to its physical substrate. *Nat. Rev. Neurosci.* **17**, 450–461, 10.1038/nrn.2016.44 (2016). 27225071 10.1038/nrn.2016.44

[23] Tononi, G. An information integration theory of consciousness. *BMC Neurosci*. **5**, 42 10.1186/1471-2202-5-42 (2004). 10.1186/1471-2202-5-42 PMC543470 15522121

[24] Vishne, G., Gerber, E. M., Knight, R. T. & Deouell, L. Y. Distinct ventral stream and prefrontal cortex representational dynamics during sustained conscious visual perception. *Cell Rep*. **42**, 10.1016/j.celrep.2023.112752 (2023). 10.1016/j.celrep.2023.112752 PMC10530642 37422763

[25] Stigliani, A., Jeska, B. & Grill-Spector, K. Encoding model of temporal processing in human visual cortex. *Proc. Natl. Acad. Sci.* **114**, E11047–E11056, 10.1073/pnas.1704877114 (2017). 29208714 10.1073/pnas.1704877114 PMC5754759

[26] Gerber, E. M., Golan, T., Knight, R. T. & Deouell, L. Y. Cortical representation of persistent visual stimuli. *NeuroImage* **161**, 67–79, 10.1016/j.neuroimage.2017.08.028 (2017). 28807872 10.1016/j.neuroimage.2017.08.028 PMC5957542

[27] Broday-Dvir, R., Norman, Y., Harel, M., Mehta, A. D. & Malach, R. Perceptual stability reflected in neuronal pattern similarities in human visual cortex. *Cell Rep*. **42**, 10.1016/j.celrep.2023.112614 (2023). 10.1016/j.celrep.2023.112614 37285270

[28] Ramírez, F. M., Cichy, R. M., Allefeld, C. & Haynes, J.-D. The Neural Code for Face Orientation in the Human Fusiform Face Area. *J. Neurosci*. **34**, 12155–12167, 10.1523/JNEUROSCI.3156-13.2014 (2014). 25186759 10.1523/JNEUROSCI.3156-13.2014 PMC6608457

[29] Kay, K., Bonnen, K., Denison, R. N., Arcaro, M. J. & Barack, D. L. Tasks and their role in visual neuroscience. *Neuron* **111**, 1697–1713, 10.1016/j.neuron.2023.03.022 (2023). 37040765 10.1016/j.neuron.2023.03.022

[30] Zheng, Z., Chen, X., Brown, T., Cousijn, H. & Melloni, L. A FAIR Workflow Guide for Researchers in Human Cognitive Neuroscience. *Preprint at PsyArXiv*10.31234/osf.io/yhj5c (2024).

[31] Loring, D. W. *et al*. Wada memory performance predicts seizure outcome following anterior temporal lobectomy. *Neurology* **44**, 2322–2322, 10.1212/WNL.44.12.2322 (1994). 7991119 10.1212/wnl.44.12.2322

[32] Wada, J. & Rasmussen, T. Intracarotid Injection of Sodium Amytal for the Lateralization of Cerebral Speech Dominance: Experimental and Clinical Observations. *J. Neurosurg.* **17**, 266–282, 10.3171/jns.1960.17.2.0266 (1960). 10.3171/jns.2007.106.6.1117 17564192

[33] Tarr, M. J. The Object Databank. *Carnegie Mellon Univ*. (1996).

[34] Willenbockel, V. *et al*. Controlling low-level image properties: The SHINE toolbox. *Behav. Res. Methods* **42**, 671–684, 10.3758/BRM.42.3.671 (2010). 20805589 10.3758/BRM.42.3.671

[35] Lepauvre, A., Hirschhorn, R., Bendtz, K., Mudrik, L. & Melloni, L. A standardized framework to test event-based experiments. *Behav. Res. Methods* **56**, 8852–8868, 10.3758/s13428-024-02508-y (2024). 39285141 10.3758/s13428-024-02508-y PMC11525435

[36] The MathWorks Inc. MATLAB. The MathWorks Inc (2019).

[37] Brainard, D. H. The Psychophysics Toolbox. *Spat. Vis.* **10**, 433–436 (2007). 9176952

[38] Jenkinson, M. & Smith, S. A global optimisation method for robust affine registration of brain images. *Med. Image Anal.* **5**, 143–156, 10.1016/S1361-8415(01)00036-6 (2001). 11516708 10.1016/s1361-8415(01)00036-6

[39] Smith, S. M. *et al*. Advances in functional and structural MR image analysis and implementation as FSL. *NeuroImage* **23**, S208–S219, 10.1016/j.neuroimage.2004.07.051 (2004). 15501092 10.1016/j.neuroimage.2004.07.051

[40] Dale, A. M., Fischl, B. & Sereno, M. I. Cortical Surface-Based Analysis: I. Segmentation and Surface Reconstruction. *NeuroImage* **9**, 179–194, 10.1006/nimg.1998.0395 (1999). 9931268 10.1006/nimg.1998.0395

[41] Yang, A. I. *et al*. Localization of dense intracranial electrode arrays using magnetic resonance imaging. *NeuroImage* **63**, 157–165, 10.1016/j.neuroimage.2012.06.039 (2012). 22759995 10.1016/j.neuroimage.2012.06.039 PMC4408869

[42] Joshi, A. *et al*. Unified Framework for Development, Deployment and Robust Testing of Neuroimaging Algorithms. *Neuroinformatics* **9**, 69–84, 10.1007/s12021-010-9092-8 (2011). 21249532 10.1007/s12021-010-9092-8 PMC3066099

[43] Groppe, D. M. *et al*. iELVis: An open source MATLAB toolbox for localizing and visualizing human intracranial electrode data. *J. Neurosci. Methods* **281**, 40–48, 10.1016/j.jneumeth.2017.01.022 (2017). 28192130 10.1016/j.jneumeth.2017.01.022

[44] Collins, D. L., Neelin, P., Peters, T. M. & Evans, A. C. Automatic 3D Intersubject Registration of MR Volumetric Data in Standardized Talairach Space. *J. Comput. Assist. Tomogr.* **18**, 192 (1994) 8126267

[45] Seedat, A. *et al*. Max Planck Institute for Empirical Aesthetics. *Cogitate iEEG Data (Dataset)*. 10.17617/1.CQYN-9A87 (2024).

[46] Seedat, A. *et al*. Max Planck Institute for Empirical Aesthetics. *Cogitate iEEG BIDS Data (Dataset)*. 10.17617/1.6qpg-gz82 (2024).

[47] Cogitate Consortium, Ferrante, O. *et al*. *Cogitate Data on XNAT*. 10.17617/1.k278-n152 (2025).

[48] Appelhoff, S. *et al*. MNE-BIDS: Organizing electrophysiological data into the BIDS format and facilitating their analysis. *J. Open Source Softw.* **4**, 1896, 10.21105/joss.01896 (2019). 35990374 10.21105/joss.01896 PMC9390980

[49] King, J.-R. & Dehaene, S. *et al*. Characterizing the dynamics of mental representations: the temporal generalization method. *Trends in Cognitive Sciences* **18**(4), 203-210, 10.1016/j.tics.2014.01.002 (2014). 10.1016/j.tics.2014.01.002 PMC5635958 24593982

[50] King, J.-R., Pescetelli, N. & Dehaene, S. Brain Mechanisms Underlying the Brief Maintenance of Seen and Unseen Sensory Information. *Neuron* **92**, 1122–1134, 10.1016/j.neuron.2016.10.051 (2016). 10.1016/j.neuron.2016.10.051 27930903

[51] Lepauvre, A. *et al*. iEEG-data-release. *Zenodo*10.5281/zenodo.13832169 (2024).

[52] Gramfort, A. *et al*. MEG and EEG data analysis with MNE-Python. *Front. Neurosci*. **7**, 10.3389/fnins.2013.00267 (2013). 10.3389/fnins.2013.00267 PMC3872725 24431986

