# Supplementary material for: Open multi-center intracranial electroencephalography dataset with task probing conscious visual perception
Source: Sci Data. 2025 May 23;12:854. doi: 10.1038/s41597-025-04833-z (PMC12102287; doi:10.1038/s41597-025-04833-z)
Supplement: Supplementary file 1 — Supplementary information [file 41597_2025_4833_MOESM1_ESM.pdf]

Supplementary

Participant ID	Native Language	EKG available	Age of Onset	Seizure Type	WADA (Language)	IQ [value, test]
CE103	English	no	27	N/A	N/A	109, FSIQ
CE106	English	yes	12	N/A	N/A	>70, FSIQ
CE107	English	yes	13	N/A	N/A	>70, FSIQ
CE108	English	yes	12	N/A	N/A	>70, FSIQ
CE109	English	no	45	N/A	N/A	>70, FSIQ
CE110	English	yes	10	N/A	N/A	>70, FSIQ
CE112	English	yes	7	N/A	N/A	>70, FSIQ
CE113	English	no	19	N/A	N/A	>70, FSIQ
CE115	English	yes	6	N/A	N/A	>70, FSIQ
CE118	English	yes	8	FIA	N/A	>70, FSIQ
CE119	Polish	no	28	FA, FIA	N/A	>70, FSIQ
CE120	English	yes	1	FBTC	N/A	>70, FSIQ
CE121	English	yes	8	BTC	N/A	>70, FSIQ
CF102	English	yes	22	FBTC	L	98, VCI; 90, POI; 83, WMI; 100, PSI
CF103	English	yes	11	FA	Predominantly L, mild R contribution	145, VCI; 96, POI; 95, WMI; 86, PSI
CF104	English	yes	13	FBTC	L	79, VCI; 62, POI
CF105	English	yes	22	FBTC	L	116, VCI; 111, POI; 102, WMI; 114, PSI
CF106	English	yes	11	FM	N/A	N/A
CF107	English	yes	23	FIA	N/A	104, VCI
CF109	English	yes	3	FIA	Predominantly L, mild R contribution	107, VCI; 86, POI; 95, WMI; 92, PSI
CF110	English	yes	2	FIA, FBTC	N/A	89, VCI; 100, POI; 83, WMI; 70, PSI
CF112	English	yes	19	FIA	L	107, VCI; 123, POI; 131, WMI; 100, PSI
CF113	English	yes	34	FA, FIA	L	100, VCI; 102, POI; 114, WMI; 102, PSI
CF116	English	yes	38	FIA	N/A	144, VCI
CF117	English	no	26	FBTC, FIA	N/A	105, VCI; 107, POI; 95, WMI; 108, PSI
CF119	English	no	36	FBTC	L	102, VCI; 98, POI; 97, WMI; 108, PSI
CF120	English	yes	44	FA, FIA	N/A	N/A
CF121	English	no	1	FIA	L	83, VCI; 77, POI; 77, WMI; 76, PSI
CF122	English	yes	14	FH	N/A	114, VCI; 96, POI;

						94, WMI; 94, PSI
CF124	Spanish	yes	15.5	FA	L	68, VCI; 88, POI; 78, WMI; 88. PSI
CF125	English	yes	20	FIA	L	N/A
CF126	English	yes	9	FIA, FBTC	L	76, VCI; 88, POI; 89, WMI; 86, PSI;
CG101	English	yes	24	FIA	N/A	115, FSIQ
CG102	English	yes	12	FA, FIA, FBTC	N/A	86, FSIQ
CG103	English	yes	1.5	FA, FIA, FBTC	N/A	77, FSIQ
CG104	English	no	30	FIA	N/A	90, FSIQ
CG105	English	yes	20	FA	N/A	15, FSIQ
CG106	English	yes	18	FIA, FBTC	N/A	110, FSIQ

***Table S1**: iEEG Patient Characteristics*

EKG – electrocardiogram channels, N/A – not applicable, L – left, R – right; FSIQ – Full Scale Intelligence Quotient, VCI – Verbal Comprehension Index, POI – Perceptual Organization Index, WMI – Working Memory Index, PSI – Processing Speed Index; FBTC – focal to bilateral tonic-clonic, FIA – focal impaired awareness, FA – focal aware seizures, FM – focal motor, FH – focal hemiclonic seizure.

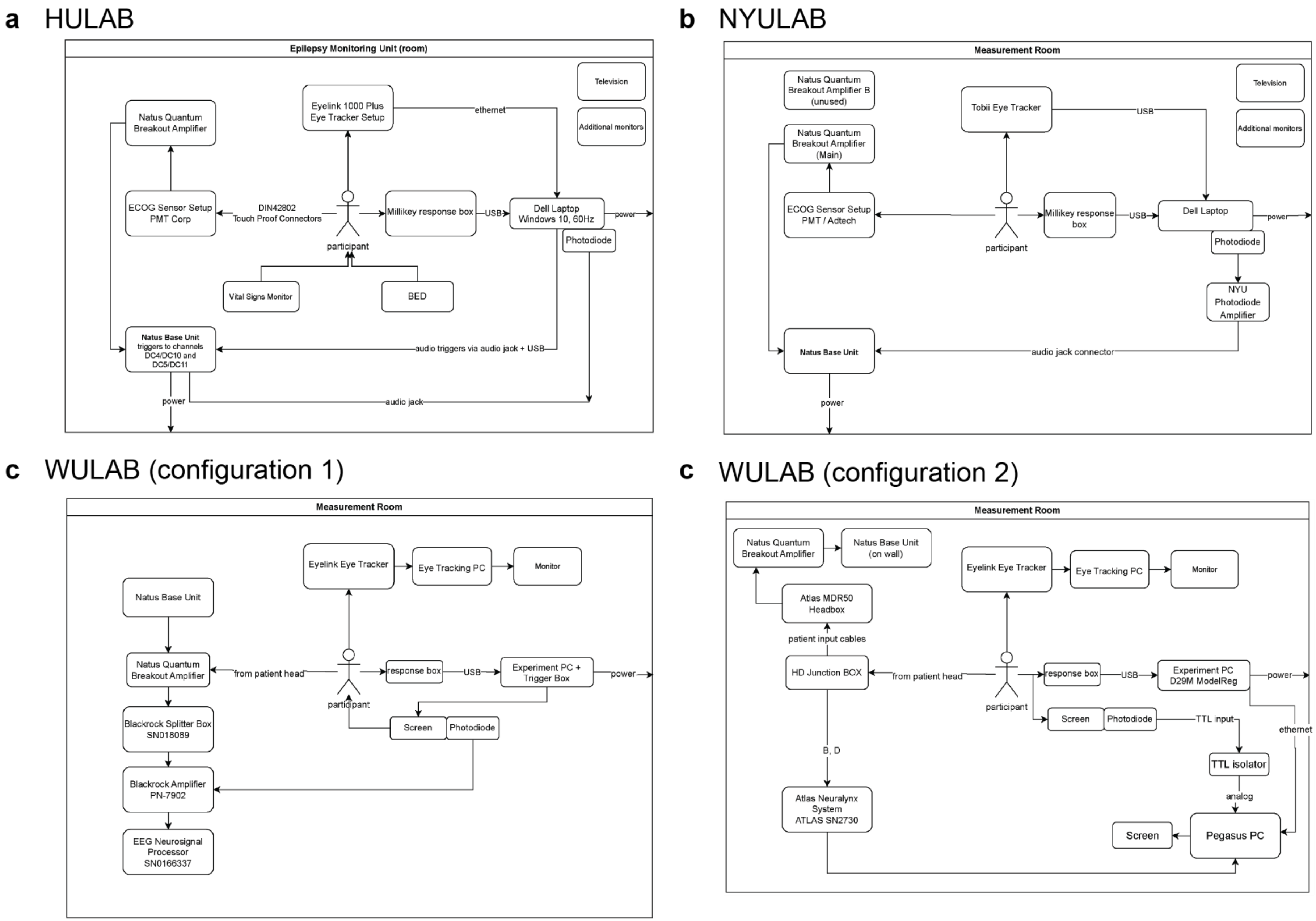

a HULAB
Epilepsy Monitoring Unit (room)
Television
Additional monitors
Natus Quantum Breakout Amplifier
Eyelink 1000 Plus Eye Tracker Setup
ethernet
ECOG Sensor Setup PMT Corp
DIN42802 Touch Proof Connectors
participant
Millikey response box
USB
Dell Laptop Windows 10, 60Hz
power
Photodiode
Vital Signs Monitor
BED
Natus Base Unit triggers to channels DC4/DC10 and DC5/DC11
audio triggers via audio jack + USB
power
audio jack
b NYULAB
Measurement Room
Natus Quantum Breakout Amplifier B (unused)
Natus Quantum Breakout Amplifier (Main)
Tobii Eye Tracker
USB
Television
Additional monitors
ECOG Sensor Setup PMT / Adtech
participant
Millikey response box
USB
Dell Laptop
power
Photodiode
NYU Photodiode Amplifier
Natus Base Unit
audio jack connector
power
c WULAB (configuration 1)
Measurement Room
Eyelink Eye Tracker
Eye Tracking PC
Monitor
Natus Base Unit
Natus Quantum Breakout Amplifier
from patient head
participant
response box
USB
Experiment PC + Trigger Box
power
Screen
Photodiode
Blackrock Splitter Box SN018089
Blackrock Amplifier PN-7902
EEG Neurosignal Processor SN0166337
c WULAB (configuration 2)
Measurement Room
Natus Quantum Breakout Amplifier
Natus Base Unit (on wall)
Eyelink Eye Tracker
Eye Tracking PC
Monitor
Atlas MDR50 Headbox
patient input cables
HD Junction BOX
from patient head
participant
response box
USB
Experiment PC D29M ModelReg
power
Screen
Photodiode
TTL input
ethernet
B, D
TTL isolator
analog
Atlas Neuralynx System ATLAS SN2730
Screen
Pegasus PC

Figure S1*: Experimental setup wiring diagrams from each of the data collection sites:* ***a.*** *Boston Children's Hospital and Brigham and Women's Hospital (HULAB),* ***b.*** *NYU Langone Health (NYULAB),* ***c. and d.*** *University of Wisconsin–Madison (WULAB). Intracranial EEG (iEEG) data were transmitted from electrodes in the head, using touch-proof connectors to the data amplifier, and then to the base unit. Simultaneously, eye-tracking data and behavioral data were collected via an eye-tracker and response box, respectively, and connected to the experimental PC. The experimental PC sent photodiode pulses through the amplifier to the base unit.* ***c.*** *WULAB (configuration 1) refers to the experimental setup for participants CG101, CG102, and CG103, whereas* ***d.*** *WULAB (Configuration 2) refers to the experimental setup for participants CG104, CG105, and CG106.*
